# Hydrothermal vent temperatures track magmatic inflation and forecast eruptions at the East Pacific Rise, 9°50’N

**DOI:** 10.1073/pnas.2510245122

**Published:** 2025-10-13

**Authors:** Thibaut Barreyre, Jean-Arthur Olive, Daniel J. Fornari, Jill M. McDermott, Ross Parnell-Turner, Kim Moutard, Jyun-Nai Wu, Milena Marjanović

**Affiliations:** ^a^Geo-Ocean, Univ Brest, CNRS, Ifremer, UMR6538, Plouzané F-29280, France; ^b^Department of Geology and Geophysics, Woods Hole Oceanographic Institution, Woods Hole, MA 02543; ^c^Laboratoire de Géologie, CNRS—Ecole Normale Supérieure, Université Paris Sciences et Lettres, Paris 75005, France; ^d^Department of Earth and Environmental Sciences, Lehigh University, Bethlehem, PA 18015; ^e^Institute of Geophysics and Planetary Physics, Scripps Institution of Oceanography, University of California, San Diego, La Jolla, CA 92037; ^f^Institut de Physique du Globe de Paris, Université Paris Cité, CNRS, UMR7154, Paris 75005, France

**Keywords:** mid-ocean ridges, hydrothermal activity, seafloor volcanic eruption, heat and energy transfer

## Abstract

Hydrothermal vent temperatures fluctuate in response to transient magmatic and tectonic activity at the axis of mid-ocean ridges (MORs) and modulate energy fluxes from the deep Earth to the ocean. Such fluctuations have thus far only been documented on time scales of minutes to years, because of the scarcity of long, continuous observations. Here, we assemble a ~35-year-long time series of exit fluid temperatures from five hydrothermal vents on the East Pacific Rise axis, between 9°46’-51’N. This dataset reveals a steady increase in maximum venting temperatures atop the central part of the axial magma lens (AML), from ~350 °C to ~390 °C between the 1991–92 and 2005–06 eruptions. Temperatures decreased back to ~350 °C shortly after the 2005–06 eruption and have been rising ever since. We interpret the temperature increase as a result of a steady decrease in upflow zone permeability caused by the steady inflation of the AML compressing the oceanic upper crust. Using laboratory-determined pressure–permeability relations, we estimate crustal pressurization rates of 0.38 MPa/y (1992–2005) and 0.33 MPa/y (post-2006), consistent with geodetic observations from 2009–2011. Decadal fluctuations in hydrothermal vent temperatures likely mimic the rate of AML pressurization, yielding valuable new constraints on the dynamics of magmatic replenishment and eruptions at MORs. Notably, this temperature time series underpinned our forecast of the April 2025 eruption at the study site.

## Significance

We document multidecadal temperature variations at a mid-ocean ridge (MOR) hydrothermal system and reveal that crustal and deep magmatic processes exert a clear control on seafloor venting temperatures. This finding has important implications, as the temperatures of hydrothermal fluids venting at the seafloor play a critical role in shaping the chemical and mineralogical environments of vent-based chemosynthetic ecosystems. We demonstrate that black smoker vent temperatures are sensitive indicators of these intertwined magmatic, tectonic, and hydrothermal processes, providing a valuable constraint on short-term magmatic activity at MORs and enhancing our ability to forecast seafloor eruptions along the global MOR system.

Hydrothermal vents at the axis of mid-ocean ridges (MORs) are the seafloor manifestation of porous convection through the brittle oceanic lithosphere, primarily fueled by the heat from axial magma lenses [AMLs; (1, 2)]. Despite the extreme temperature of the underlying magma (~1,200 °C), the temperature of the hydrothermal fluids is capped at ~400 °C to 450 °C because of the thermodynamic properties of seawater. Within this temperature range, gradients of fluxibility, a measure of the efficiency of heat transport, are maximized (3, 4). The thermo-chemical output of MORs is leveraged by unique chemosynthetic organisms and, through trophic relationships, sustains entire deep-sea ecosystems (e.g., 5).

**Fig. 1.**
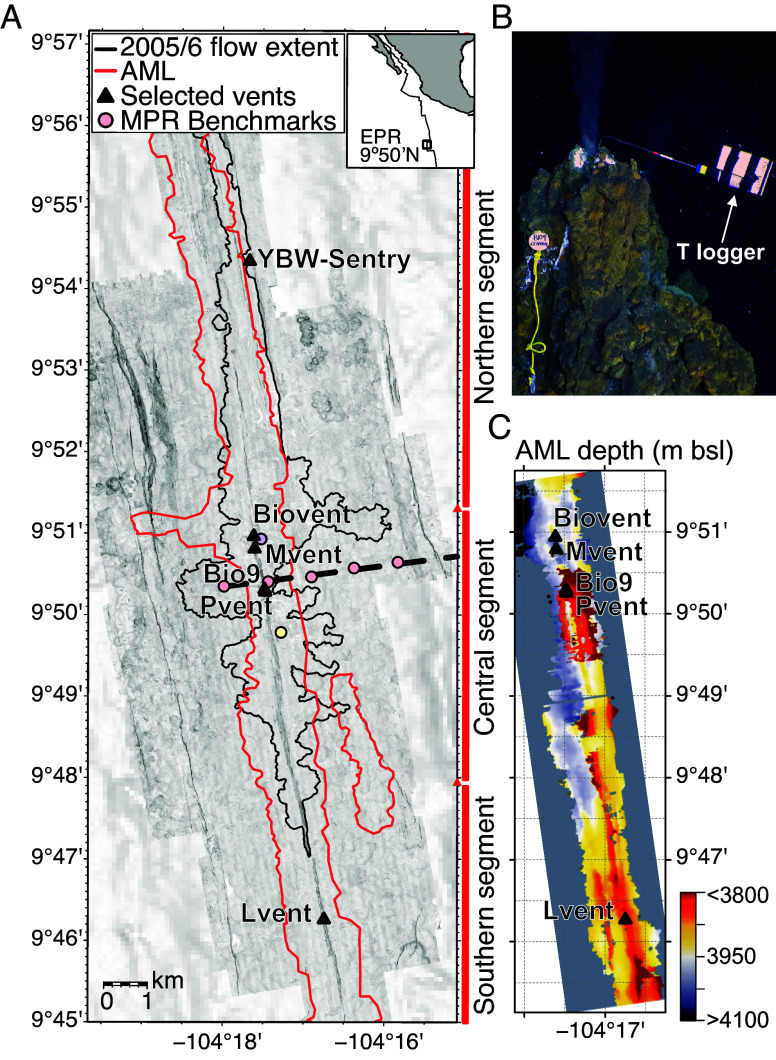
Location of hydrothermal vent temperature and geodetic station data: (*A*) 1-m resolution bathymetric data collected by AUV *Sentry*; black outline is 2005–2006 lava flow extent (14); the red outline is the extent of the AML (15); the black dashed line is geodetic line shown in Fig. 4*A*, filled circles are the 8 mobile pressure recorder (MPR) benchmarks (16); and black triangles are selected active high-temperature hydrothermal vents. Latitudinal extent of the magmatic segments (17) is also shown at right. The inset in the upper right corner shows general geographic location. (*B*) Photograph of the Bio9 vent orifice showing a high-temperature logger deployed by HOV *Alvin* monitoring long-term black smoker vent temperature every 10 min. (*C*) Axial Magma Lens (AML) depth in meters below sea-level from (15). AML is ~250 m shallower beneath Bio9 and P vents (central vents) than beneath M vent and Biovent (northern vents), and ~50 m shallower than beneath L vent (southern vent).

MOR environments are inherently dynamic. They regularly experience spreading events such as seafloor volcanic eruptions fed by the intrusion of meter-wide dikes (e.g., 6, 7). At a fast-spreading MOR like the East Pacific Rise (EPR, Fig. 1) at 9°50’N (~10 cm/y full rate), such events are expected to occur every ~10 to 15 y on average. While the immediate response of hydrothermal circulation to sudden events has previously been explored from discrete observations and modeling of vent temperature time series (e.g., 8–12), little is known about how hydrothermal activity fluctuates over decadal time scales. These longer time scales are key, as they include the time between spreading events when AMLs are being replenished and extensional stresses build up at the ridge axis (13). This knowledge gap is largely due to the lack of long, continuous time series of in situ vent fluid temperatures, which require instruments that can withstand the intense heat and pressure at MOR axial depths and the caustic chemistry of vent fluids.

Here, we report the longest record of in situ vent fluid temperatures (35 y) from five deep-sea hydrothermal vents at the EPR between 9°46’N and 9°51’N (Fig. 1). This record reveals the degree to which decadal variations in magmatic and hydrothermal activity are intertwined.

## EPR 9°46’N–9°51’N Vent System

Hydrothermal vents along the EPR between 9°46’N and 9°51’N are located on a volcanically active, fast-spreading ridge segment that has experienced three well-documented eruptive phases ~15 to 20 y apart, in 1991–1992, 2005–2006 (6, 18–21), and most recently in 2025 (22). The earlier two eruptions and their intereruption periods were investigated during numerous field experiments that included long-term exit-fluid temperature monitoring and fluid sampling (e.g., 14, 18, 23–30). Additionally, continuous seismicity monitoring was carried out prior to and during the 2005–2006 eruption cycle (6, 31), and active seafloor uplift was measured using seafloor geodetic instruments (pressure gauges) from 2009 to 2011 (16), both of which suggested inflation of a shallow magmatic reservoir.

Melt beneath the EPR crest between ~9°N and 10°N is stored in a nearly continuous, <50 to 100 m-thick AML, located ∼1.5 km below the axis (15, 17, 32, 33), that is heating fluids in a reaction zone above the magma body (34). Focused hydrothermal circulation within the narrow (≤100 m wide) axial summit trough (AST) along this portion of the EPR crest (35) is thought to be driven by continuous AML replenishment (36) and repeated diking events located beneath or immediately proximal to the AST (24). The focused high-temperature hydrothermal vents along the EPR axis between 9°46’ and 9°51’N are typically grouped as a southern (L vent), central (P and Bio9 vents), and northern (M and Biovent vents) clusters (25) (Fig. 1). These vents were impacted to varying degrees by the two eruption phases in 1991–1992 and 2005–2006, which altered the hydrothermal plumbing system associated with the fracture and fissure systems within and beneath the AST along the axis (30). Eruptions are thought to reset the deviatoric stress field at the ridge axis (37), thereby altering the permeability of the hydrothermal plumbing system (30, 38).

L vent is located on the southern segment of the AST between 9° 45.8’-46.6’N (14), above an area where the melt lens is ~50 m deeper than the shallowest AML section, located between ~9°48’N-50’N (15). The central Bio9 and P vents lie above a small magma lens emplaced within the upper crust ~150 m above the main AML (15), collocated with the shallowest bathymetry along the EPR crest in the central segment between 9°50.2’N and 9°50.6’N. These hydrothermal vent areas are the best-studied and sampled chimneys since *Alvin* diving began at this study site in March 1991 (e.g., 18, 25). Bio9 and P vents (Fig. 1) are centered over the most active region of the magma system (17, 39, 40), while M and L lie farther from the center of subcrestal magmatic activity, and Biovent is positioned at the northern end of the AST central segment.

## A ~35-y Time Series of Vent Temperatures From Multiple Sites

**Fig. 2.**
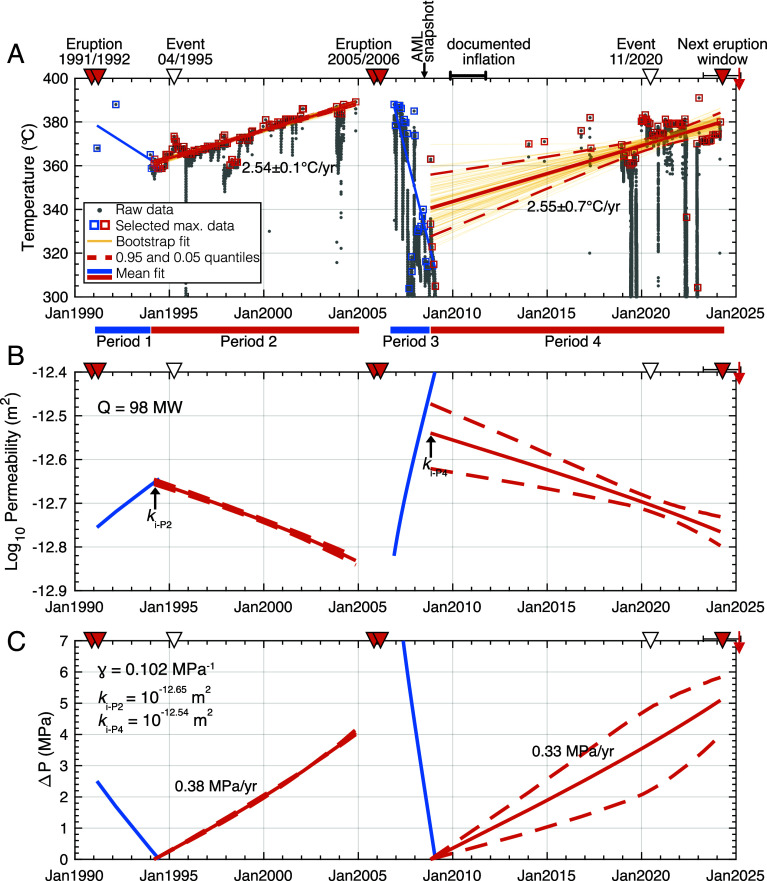
Vent temperature, modeled crustal permeability, and overpressure: (*A*) Maximum exit fluid temperature, Bio9 vent. Gray dots/squares are raw/filtered data, respectively; blue/red lines are fits for post/pre-eruption periods, respectively. The black arrow denotes 3-D seismic survey (15); period of documented inflation noted (16). Red/white inverted triangles are volcanic eruptions/anomalous hydrothermal events, respectively (e.g., 8, 23); red arrows show most recent seafloor eruption in 2025 (22); yellow lines are fits to individual bootstrap data; the solid red line is the primary fit (i.e., average of all bootstrap estimates) for temperature vs. time for Bio9 during the 1991–1992 and 2005–2006 eruptions; and red dashed lines are defined quantiles (i.e., 95% of estimated fits are within the quantified red dashed error bands). (*B*) Temperature-derived permeability computed (*Methods*) (41) assuming heat flux = 98 MW (42); arrows show initial permeabilities for periods 2 and 4. (*C*) Permeability-derived overpressure (*Methods*) (43).

We have compiled a 35-year-long time series of hydrothermal vent temperatures (including discrete measurements made during fluid chemistry sampling) for each of these five vent areas (Bio9 in Fig. 2, and Biovent, M vent, P vent, and L vent in *SI Appendix*, Fig. S1). The maximum temperature (i.e., proxy for the endmember, undiluted hydrothermal fluid) of the EPR black smokers is extracted from these long time series (*Methods*) and reveals temporal trends that suggest a strong linkage with AML replenishment–eruption cycles (Fig. 2 and *SI Appendix*, Fig. S1). Between eruptions, temperature increases on the order of tens of °C occur on decadal time scales, while posteruption decreases in temperature occur within a few years (Fig. 2). The maximum temperature reached across the system is ~390° ± 5 °C, which corresponds to the thermodynamic limit defined by the critical point of seawater at the seafloor depth of 2.5 km (∼250 bar). The compiled record allows us to compare the rate of temperature change during two intereruption periods, using the mean slope obtained from bootstrapped samples (*Methods* and *SI Appendix*, Fig. S2). First, shortly after the 1991–92 eruption and before the next eruption in 2005–06, maximum vent temperatures increased at a rate of 2.54 ± 0.1 °C/y (where the uncertainty is constrained by the dispersion or SE of the slope). Second, from 2006 until 2024, we obtain a rate of 2.55 ± 0.7 °C/y. Our error estimate is larger for this second period due to the ~5-y data gap between January 2009 and January 2014 when no deep submergence field programs took place at the study area. Despite this uncertainty, the rates of temperature increase over time are strikingly similar between the two eruption periods (within the error limits), but the basal temperature (departure temperature for the increase/build-up) is notably lower for the second period (~310° to 330 °C in January 2009) than it was in January 1994 (~360 °C).

Furthermore, the temperature time series data also captured multiple transients (that is, departures from the increasing decadal trend), similar to observations related to the 1995 event at the Bio9 vent (8, 23), some of them affecting multiple sites (*SI Appendix*, Fig. S3), with recurrence intervals as short as ~2 y. These transients, superimposed on the long-term temperature increase trend, have been modeled as noneruptive diking events (11) and could play an important role in accommodating plate separation, maintaining/enhancing hydrothermal convection, and modulating magma-induced tectonic activity (44).

Based on long-term increases in Bio9 exit-fluid temperature trends over the past ~15 y (period 4; Fig. 2), vents on the EPR axis between ~9°46’-51’N appear to be experiencing temperature conditions in early 2025 that are similar to those which preceded eruptive phases in 1991–1992 and 2005–2006, suggesting the potential for an eruption phase during the next few years. On 29 April 2025 (while this paper was undergoing peer review), a seafloor volcanic eruption was directly observed by scientists diving in HOV *Alvin* (AT50-36 expedition) at 9°50’N EPR (22). This agreement between forecast and observation demonstrates that decadal trends in vent temperatures hold valuable information related to the EPR’s magmatic cycles.

## Inferring Decadal Changes in Subseafloor Permeability

**Fig. 3.**
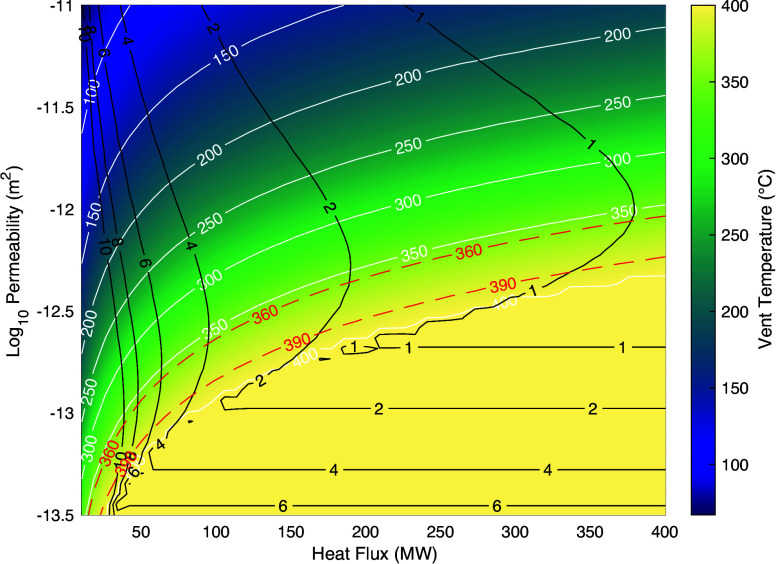
Modeled hydrothermal fluid vent temperature (°C) as a function of heat (MW) transported advectively (over an area of π*r^2^, where r is the radius of the upflow zone, 50 m radius is used in this model) and crustal permeability (m^2^) within the zone of fluid flow. Grid was computed from Eq. (**1**) using a value of r = 50 m (half width of hydrothermal plume) and for fluid properties at 30 MPa. White solid lines are vent temperature contours. Red dashed lines indicate the EPR Bio9 vent temperature range during period 2, where exit fluid temperatures increased from 360 °C to 390 °C. Black solid lines are hydrothermal fluid advection times (years), corresponding to the interval required for the hydrothermal fluid to reach the seafloor from the reaction zone (i.e., base of the convection cell).

An increase in vent temperatures can be explained by a combination of an increasing input of heat from the underlying magmatic system and a decrease in crustal permeability. A decrease in crustal permeability under constant heat input reduces the fluid mass flow rate, which leads to the fluid receiving more heat per unit mass and reaching hotter temperatures (Fig. 3 and *SI Appendix*, Fig. S4; *Methods*) (41). Several studies have attempted to quantify the integrated hydrothermal heat output from the EPR 9°50’N study area. These estimates range from 42 MW for vent orifices at Bio9 (45) to 98 MW for the combined Bio9 and P vent fields, assuming that they are fed by a common discharge zone ~100 m in diameter (42). Accounting for low-temperature diffuse venting, a value of 160 MW was calculated for a region between Tubeworm Pillar and Bio9 vents (~1.5 km distance) (2), and an estimate of 325 MW was obtained for a ~2 km section of ridge axis from Biovent near 9°51’N to Ty/Io vents (south of P vent) (42). Fig. 3 shows that for an upflow zone to transport ~100 MW of heat with fluid temperatures between 360 °C and 390 °C, its average permeability must be ~2 × 10^−13^ m^2^ (*Methods*), consistent with prior estimates (2, 46, 47). Alternatively, a heat output of ~300 MW requires a permeability of ~5.6 × 10^−13^ m^2^ (Fig. 3).

The steady increase in vent temperatures between eruptions (Fig. 2*A*) could be explained by a steadily increasing supply of magmatic heat from below. Driving a 30 °C increase in vent temperatures solely by changes in heat flow (*Q*) requires the heat input/output to almost double (Fig. 3 and *SI Appendix*, Figs. S5 and S6*B*). This result implies a decadal increase in magma supply to the shallow AML (36), likely manifesting as accelerating rates of seafloor uplift due to faster rates of magmatic replenishment. To our knowledge, even at the highly magmatically active Axial Seamount site (e.g., 48, 49), rates of seafloor uplift generally hold steady or decelerate on intereruption time scales (5 to 10 y). In the following analysis, we therefore focus on the other endmember explanation, in which the heat input/output remains uniform (i.e., magma is being supplied to the AML at a constant rate), and the average crustal permeability steadily decreases. This scenario is more plausible since the effective permeability of hydrothermal systems is known to fluctuate even on monthly time scales (30). Assuming the crustal upflow zone beneath Bio9 transports 98 MW of heat (42), we construct a time series of the average crustal permeability (Fig. 2*B*) that explains the observed trends in vent temperatures (Fig. 2*A*). Assuming a different heat output would shift these permeability values up and down, but retains the relative trends (Fig. 3 and *SI Appendix*, Fig. S7): Average upper crustal permeability appears to steadily decrease by ~0.2 log units (i.e., divided by ~1.6) per decade between documented EPR eruptions, and to reincrease by up to ~0.3 log units (i.e., double) over a few years following each eruption.

An increase in shallow crustal permeability was observed following the January 2006 diking event using tidal modulations of vent temperatures (30). Even if permeability were to change instantaneously across the entire upper crust, vent temperatures would still require a finite amount of time (*τ*) to adjust to the new convective flow conditions (*Methods*). Fig. 3 shows that *τ* ranges from ~4 to ~1 y depending on the assumed heat output *Q* ~100 to ~300 MW, respectively. This result is consistent with the duration of periods 1 and 3 in Fig. 2*A*. It is therefore possible that the average permeability of the upper crust decreases steadily between eruptions and suddenly increases during dike intrusion events, with vent temperatures taking a few years to respond following the disruptions in crustal fluid pathways caused by dike intrusion. Fluctuations of vent temperatures on decadal time scales can thus be considered proxies for changes in subseafloor hydrology to depths equal to that of the AML.

## Linking Permeability Changes to Magmatic Inflation

Permeability of the oceanic crust is primarily controlled by cracks and pores of all sizes providing connected pathways to channel hydrothermal fluids (50). If stresses are large enough to overcome the brittle strength of the material, crustal deformation may affect permeability by prompting the growth of new crack surfaces and modifying pore connectivity (51). Small elastic strains can also significantly alter permeability by changing the typical aperture of cracks (52). Experimental studies of this phenomenon have yielded nonlinear relations between (effective) confining pressure and permeability, either exponential (43, 53), or power-law type (54, 55). The estimates shown in Fig. 2*B* suggest exponentially decreasing permeability during periods 2 and 4, consistent with a linearly increasing effective pressure *P* if the pressure–permeability relation is exponential.

If we assume the upper oceanic crust has hydromechanical properties similar to those of Kola basalts (see 43, and *Methods*), the permeability trends of Fig. 2*B* require an increase in pressure of 4 MPa over 10.5 y for period 2, and 5 MPa over 15 y for period 4. This pattern corresponds to mean crustal pressurization rates of 0.38 MPa/y and 0.33 MPa/y for periods 2 and 4, respectively.

**Fig. 4.**
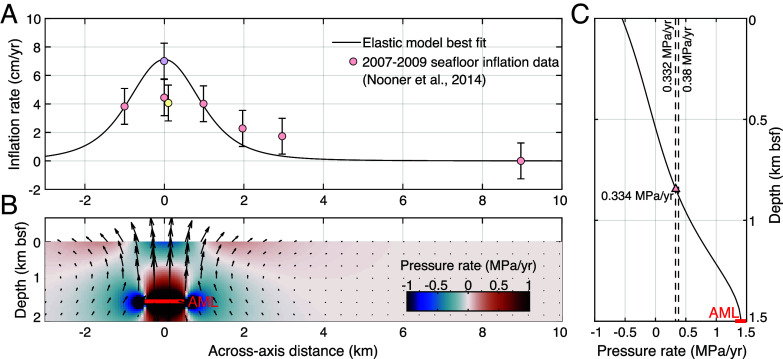
Measured and modeled seafloor inflation. (*A*) Colored dots are seafloor uplift recorded by pressure gauges across the EPR axis in 2007–2009 (16), (shading reflects station locations, see Fig. 1*A*); the black line is uniform elastic half space model (59) best fit determined in this study. (*B*) Elastic model (across-axis cross-section) of the opening of a horizontal, 1,000 m wide sill (red segment) 1.5 km below seafloor, at 0.18 m/y. This amounts to 180 m^3^ of melt delivered to the AML every year, per meter along the ridge axis, which is close to the time-averaged flux required to build a ~150 m-thick layer 2A. Colors mark associated changes in rock pressure in MPa/y. (*C*) Depth-averaged overpressure above the opening of the magma sill (i.e., mean crustal overpressure above magma sill) is 0.3339 MPa/y and shown as a pale red triangle. Both derived overpressure rates for periods 2 (0.38 MPa/y) and 4 (0.332 MPa/y) in Fig. 2*C* are shown by black dashed lines. Note the good agreement between pressure rate estimates derived from elastic model (59), constrained by in situ geodetic data (16), and pressure rates calculated from models using time-series temperature trends.

A plausible cause of this steady compression of the upper crust is steady overpressurization of the AML as it gets replenished with magma between eruptions (31, 38, 56). Distributed seafloor uplift at the EPR 9°50’N site was documented between 2009 and 2011 (Figs. 1*A* and 4) (16) and was attributed to the inflation of either a point-source (57) at 2.7 km below the ridge axis or a shallower (1.5 km bsf) axisymmetric penny-shaped crack inflation (58) source (3 km wide). Importantly, the magma supply rate they inferred was deemed sufficient to source lava flows comparable in area and volume to the 2005 to 2006 eruptions (14, 20) by the year 2026 (16).

We find that a horizontal mode-I dislocation in an elastic half-space (i.e., an opening horizontal sill; 60) also provides a reasonable fit to the seafloor uplift data if we assume the sill coincides with the seismically imaged AML (1.5 km deep, 1 km wide) and opens at a rate of 0.18 m/y (Fig. 4*A*). We note that this 2-D model fails to capture along-axis gradients in seafloor uplift, which suggests that magmatic inflation is variable along-axis. This result may help explain the relatively short length (~500 to <1,000 m) of mapped axial eruptive fissures that sourced the 2005–2006 eruptions (20, 61, 62), as well as the along-strike discontinuities in the AML. This model also slightly underestimates the cross-axis extent of seafloor uplift, either because the width of the sill is underestimated, or more plausibly because it ignores the viscous properties of the sub-AML mush zone (e.g., 63) which would widen the seafloor deformation pattern. Nonetheless, this simple model provides helpful insight into how the upper crust may respond to AML inflation (Fig. 4*B*). We expect strong pressurization of the base of the upper crust up to a kilometer above the AML, and a slight depressurization of the shallow crust caused by the free surface effect (Fig. 4*C*). Averaging the pressure change across the vertical extent of hydrothermal up-flow zones (AML to seafloor) yields a pressurization rate of 0.334 MPa/y (red triangle, Fig. 4*C*), which is consistent with the pressurization rates inferred from vent temperature variations (via modeled permeability changes, Fig. 2).

## Heterogeneous Crustal Permeability and Stress

**Fig. 5.**
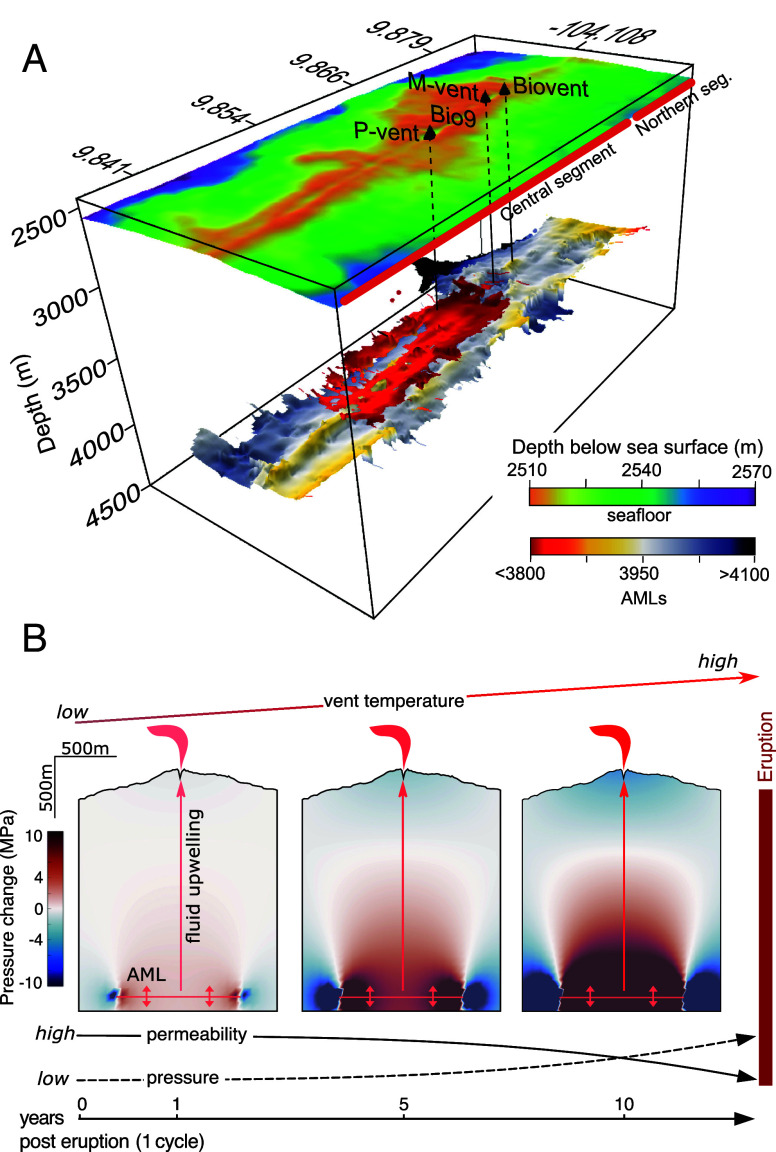
Conceptual model of intertwined magmatic and hydrothermal processes. (*A*) 3D view of AMLs and bathymetry at the EPR 9°50’N (15). Note the shallow magma body beneath Bio9 and P vents (red colored areas), which are ~300 m shallower than the main magma body (gray- to yellow-colored areas). (*B*) Across-axis modeled cross-sections of inferred crustal pressure changes above the AML at 1, 5, and 10 y, and associated relative changes in vent temperature, crustal pressure, and permeability experienced by high-temperature hydrothermal vents before, during, and after a volcanic eruption. Stress and overpressure build up before an eruption causes a decrease in permeability within the hydrothermal plumbing system, resulting in an increase in vent temperature. When diking and eruption occur, crustal stress and overpressure are released, resulting in a permeability increase that in turn causes the exit fluid temperature of a hydrothermal vent to drop.

Differing temperature trends observed at vent sites other than Bio9 (*SI Appendix*, Fig. S1) likely reflect the heterogeneous permeability and stress fields imparted by the complex three-dimensional geometry of the EPR 9°50’N hydrothermal system, AML roof topography, and spatial gradients in magmatic inflation rates (15). Bio9 and P vents exhibit the largest rate of temperature change (and thus inferred permeability and pressure), likely due to their location centered directly above a rapidly inflating portion of the AML where it is shallowest along the segment between 9°N and 10°N (Fig. 5). In contrast, M and L vents, located above the deepest portion of the AML, experience lower rates of change in temperature, permeability, and pressure (*SI Appendix*, Figs. S1 and S8). Biovent, which displays inverted temperature, permeability, and pressure tends, is located at the northern termination of the AST’s central segment where AML inflation could plausibly induce crustal depressurization (Fig. 5).

During the immediate posteruption periods 1 and 3 (Fig. 2), when vent temperatures decrease rapidly, the associated AML deflation could be instantaneous, but the 3-year trend could be a manifestation of the hydrothermal response time as demonstrated in Fig. 3, that is, the time necessary for the temperature change to advect along the entire crustal upflow zone.

Sustaining high-temperature venting requires consistently elevated heat flux via magmatic emplacement into the shallow crust, which at times leads to eruptions on the seafloor. Magmatic emplacement events will alter stress conditions in the crust, with compression above the AML resulting in locally decreased permeability, and decompression on the flanks of the AML resulting in locally increased permeability (Figs. 4 and 5). Modeling the time-series of vent fluid temperatures at each of the monitored vents between 9°46’N and 9°51’N supports the idea that an increase in crustal stresses caused by pre-eruption inflation causes a decrease in permeability within the hydrothermal plumbing system. The net impact of these interconnected phenomena is an increase in vent exit-fluid temperatures. When diking and eruption occur, crustal stress and magmatic overpressure are released, resulting in a permeability increase that causes the vent temperatures to drop.

Black smoker vent temperatures are shown to be sensitive indicators of these intertwined magmatic, tectonic, and hydrothermal processes, yielding a valuable constraint on short-term magmatic activity at MORs, potentially expanding our ability to forecast seafloor eruptions along the global MOR system.

## Methods

### Exit Fluid Vent Temperature.

Our in situ vent fluid temperature database includes both discrete measurements collected in conjunction with vent fluid chemistry sampling and continuous time series data obtained using high-temperature loggers developed and maintained by the Woods Hole Oceanographic Institution’s MISO Facility (see: https://www2.whoi.edu/site/miso/multidisciplinary-instrumentation-in-support-of-oceanography/high-temperature-loggers/). The resulting compiled vent temperature database is publicly accessible as an open-source resource (*Data, Materials, and Software Availability*).

Our time series analysis was designed to ensure that the most significant temperature trends were captured. The highest temperature is considered the most representative of the end-member fluid, as lower temperatures are often influenced by measurement issues, such as the temperature not being taken directly in the vent, local mixing with seawater, or high-frequency processes like turbulence. Furthermore, there is notable high-frequency short-term variability in the raw data, as well as gaps in data coverage due to reduced field activities at the EPR, e.g., during the 2009–2014 period. Since our study focused on long-term vent temperature trends, we removed short-term fluctuations in vent effluent temperature (i.e., shallow subsurface high-frequency variability) and reduced the clustering effect observed in the raw data. This was done using a block maxima approach (64), which applies a nonoverlapping sliding window that selects only the highest temperature value within each one-month window (shown as squares in Fig. 2).

We tested a range of time windows (15 to 60 d with an increment of 5 d) and found that a 30-d window provided the best trade-off between temporal resolution and a minimal slope estimate error, given the variation in sampling intervals and record lengths found in the data compilation (*SI Appendix*, Fig. S2). This sliding window approach enabled the identification of peak temperature values across the integrated dataset.

Finally, temperature trends (solid orange lines in Fig. 2) were determined using linear polynomial regressions, with robustness ensured through a bootstrap resampling technique that resampled the dataset 1,000 times (individual yellow lines shown in Fig. 2*A*). This approach allowed for a comprehensive assessment of the variability and reliability of the estimated slopes.

### Deriving Modeled Permeability.

The heat flux (*q*, W/m^2^) carried by a Darcyan upflow through a homogeneously permeable crust (with permeability *k*) can be written as

[1] $$q=kg\frac{\left(\rho_{0}-\rho_{*}\right)\rho_{*}\left(h_{*}-h_{0}\right)}{\mu_{*}}\;=\;kgF,$$

where *g* is the acceleration of gravity, $\mu_{*}$ is the dynamic viscosity of the upwelling fluid, $\rho_{0}$ and $\rho_{*}$ denote the density of cold seawater and of the hot upwelling fluid, respectively; $h_{0}$ and $h_{*}$ denote the specific enthalpy of cold seawater and of the upwelling fluid, respectively (that depends on the fluid temperature *T* at given pressure *P*); and *F* is the fluxibility of the upwelling fluid. Eq. (**1**) can be recast as a function *F* (3). Fluxibility is known to increase sharply with temperature as the fluid approaches criticality near 400 °C. Eq. (**1**) can thus be inverted to yield the upwelling fluid temperature that can transport a given heat flux for a given crustal permeability. This approach is illustrated in Fig. 3 (*SI Appendix*, Figs. S5 and S6), where the heat flux, *Q*, is integrated over the cross-sectional area of a cylindrical up-flow zone (*Q* = π*R*^2^*q*, assuming *R* = 50 m, e.g., 47). The thermodynamic properties of cold ocean water are calculated at *T_0_* = 2 °C and 25 MPa (seafloor pressure), while those of hot upwelling fluids (and their viscosity) are calculated at 30.145 MPa for a range of possible temperatures. Calculations rely on the IAPWS IF97 standard for pure water and Holzbecher’s viscosity model (65).

The response time of vent temperature to changes in convective flow conditions can be estimated by the characteristic time, *τ*, necessary for any temperature anomaly to travel from the heat source up to the seafloor (distance *H* at upflow velocity *U*), where

[2] $$\tau =\frac{H}{U}=\frac{H\mu_{*}}{gk\left(\rho_{0}-\rho_{*}\right)}.$$

### Deriving Modeled Pressure.

We rely on an exponential model relating confining pressure and permeability (43),

[3] $$k=k_{0}e^{-\gamma P}.$$

where $k_{0}$ is the reference permeability of the material at zero confining (effective) pressure (e.g., at the seafloor), and γ is a pressure-sensitivity parameter (53), where γ = 0.102 MPa^−1^ following experiments on Kola basalts (43). Other γ values were used for sensitivity tests and are shown in Supplemental Material (*SI Appendix*, Fig. S7*B*). From Eq. (**3**), the relative change in effective pressure ΔP required to drive a change in permeability from *k_i_* to *k_f_* is

[4] $$\Delta P=\frac{-1}{\gamma }ln\left(\frac{k_{f}}{k_{i}}\right)$$

These pressure changes are plotted in Fig. 2*C*, using the start of periods 2 and 4 as reference points.

### Modeling Axial Magmatic Inflation.

We model AML inflation as the opening of a 1,000-m wide horizontal dislocation located at a depth of 1,500 m below the free surface of a 2-D elastic half-space. We assume a Young’s modulus of 30 GPa and Poisson ratio of 0.25 (66). The calculations are carried out with an algorithm for the calculation of exact displacements, strains, and stresses (59) and closely match an analytical model of deformation of a uniform half-space (60). The depth and horizontal extent of the AML primarily control the cross-axis extent of seafloor uplift (Fig. 4*A*), while peak uplift rate scales linearly with the prescribed AML opening rate. Neglecting along-axis gradients in magmatic inflation rate for simplicity, we index our AML opening rate on the maximum rate of seafloor uplift (~7 cm/y, Fig. 4*A*), which was observed ~1 km to the north of the cross-axis geodetic transect (Fig. 1*A*, 16). Pressure rates are calculated as minus the average of the three diagonal stress components (Fig. 4 *B* and *C*). Here, we implicitly assumed that crustal pressure changes induced by magmatic inflation should dominate those potentially caused by plate divergence. An order-of-magnitude estimate assuming 10 cm/y of stretching distributed across a ~50 km wide cross-axis distance (67) yields a divergence-induced decrease in crustal pressure on the order of 0.05 MPa/y, potentially less if the elastic lithosphere is less compressible. This value is significantly lower than the pressurization rate caused by magmatic inflation (Fig. 4), supporting our decision to neglect tectonic stretching to first order in our analysis.

## Supplementary Material

Appendix 01 (PDF)

## Data Availability

The datasets generated during and/or analyzed during the current study are available in the MGDS repository [DOIs: http://dx.doi.org/10.60521/332402 (68), http://dx.doi.org/10.60521/332403 (69), http://dx.doi.org/10.60521/332405 (70), and http://dx.doi.org/10.60521/332406 (71)].
