# Supplementary material for: Hydrothermal vent temperatures track magmatic inflation and forecast eruptions at the East Pacific Rise, 9°50’N
Source: Proc Natl Acad Sci U S A. 2025 Oct 13;122(42):e2510245122. doi: 10.1073/pnas.2510245122 (PMC12557535; doi:10.1073/pnas.2510245122)
Supplement: Supplementary file 1 — Appendix 01 (PDF) [file pnas.2510245122.sapp.pdf]

PNAS

Supporting Information for
Hydrothermal vent temperatures track magmatic inflation and forecast eruptions at the East Pacific Rise, 9°50'N

Thibaut Barreyre, Jean-Arthur Olive, Daniel J. Fornari, Jill McDermott, Ross Parnell-Turner, Kim Moutard, Jyun-Nai Wu, and Milena Marjanović

Corresponding author: Thibaut Barreyre
Email: thibaut.barreyre@univ-brest.fr

This PDF file includes:

Figures S1 to S8
Supporting text
Table S1
SI References

Figures

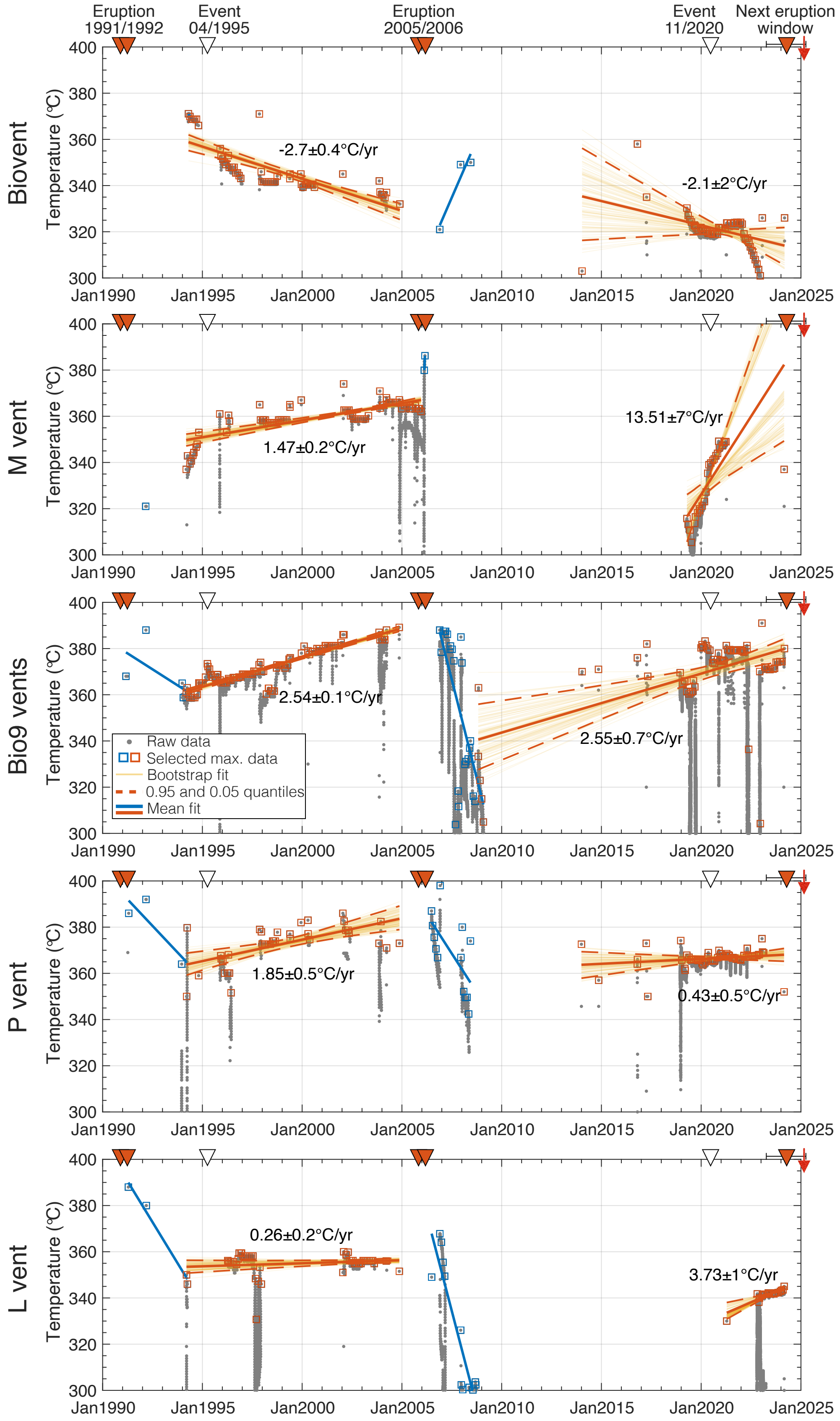

Eruption 1991/1992
Event 04/1995
Eruption 2005/2006
Event 11/2020
Next eruption window
Biovent
M vent
Bio9 vents
P vent
L vent
Temperature (°C)
-2.7±0.4°C/yr
-2.1±2°C/yr
1.47±0.2°C/yr
13.51±7°C/yr
2.54±0.1°C/yr
2.55±0.7°C/yr
1.85±0.5°C/yr
0.43±0.5°C/yr
0.26±0.2°C/yr
3.73±1°C/yr
Raw data
Selected max. data
Bootstrap fit
0.95 and 0.05 quantiles
Mean fit
Jan1990
Jan1995
Jan2000
Jan2005
Jan2010
Jan2015
Jan2020
Jan2025

Fig. S1. 30+ years of exit fluid vent temperatures (from north to south), at Biovent, Mvent, Bio9 vent, P vent and L vent. Raw data (discrete measurements + time-series from 1991 to 2024) are shown by gray dots and filtered values (i.e., maximum temperature data in a given time window, are shown by squares, blue for post-eruption times and red for pre-eruption intervals). Red/white inverted triangles are volcanic eruptions / anomalous hydrothermal events, respectively (e.g., 1, 2); red arrows show most recent seafloor eruption in 2025 (3); yellow lines are fits to individual bootstrap data; solid red line is the primary fit (i.e., average of all bootstrap estimates) for temperature vs. time for Bio9 during the 1991-1992 and 2005-2006 eruptions; and red dashed lines are defined quantiles (i.e., 95% of estimated fits are within the quantified red dashed error bands). Note that the slopes for period 2, the best constrained by data for all sites, are the highest at the Bio9 vent, which was the locus of the last two eruptions, and that they decrease to the south and north from the 9° 50'N area. Biovent, located at the end of the magmatic central segment (Fig. 1) displays an opposite temperature dynamic likely reflecting opposite permeability and pressure changes in the crust at the end of magmatic segment vs. Bio9 which is near the center of the main magmatic segment.

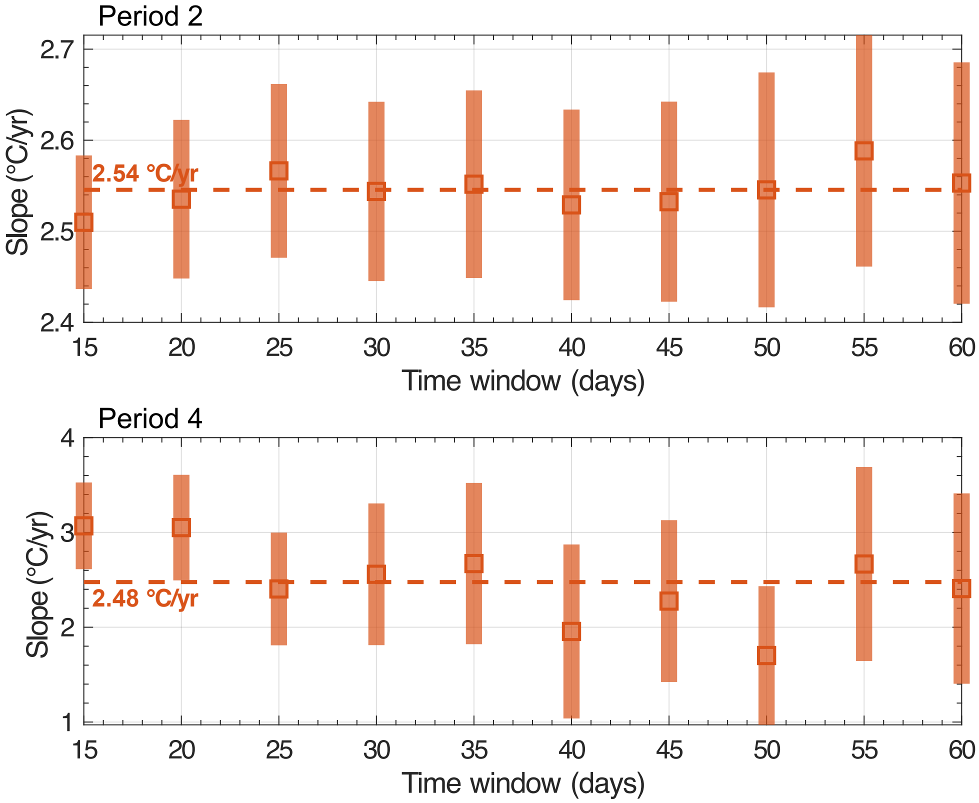

Fig. S2. Calculated temperature trend slopes (°C/yr) for periods 2 and 4 (corresponding to periods of increasing exit fluid temperature post eruption) over different time window lengths when carrying out the analysis. The modeling employed selected maximum temperature data in a given time window, then fitted each individual bootstrap T vs. time dataset, to calculate the main fit (average of all bootstrap fit estimates), which is shown as a red square and the standard deviation (2 sigma) is shown as vertical pale red bars.

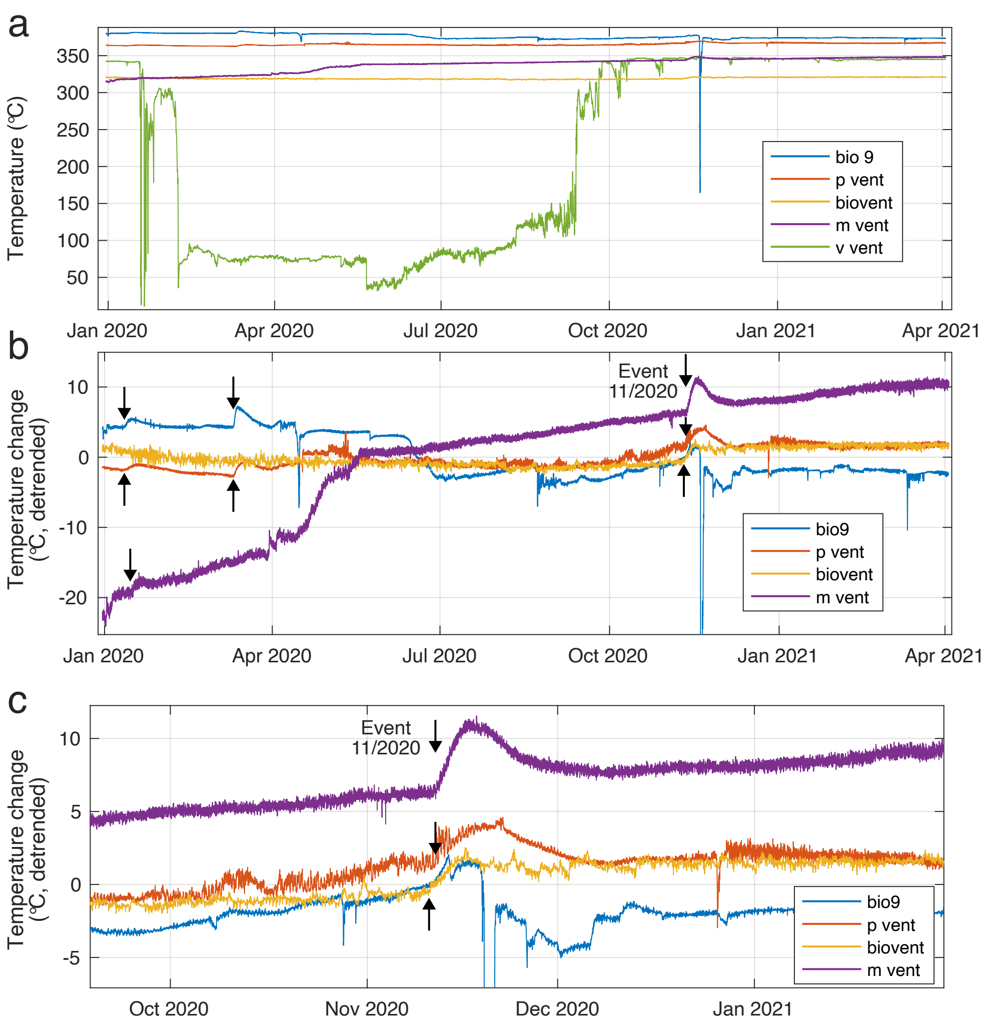

Fig. S3. Temperature records from the high-temperature loggers deployed in 2019 and recovered in 2021. (a) Raw temperature records, different vents/mounds are shown by different colors. (b) post-processed temperature records for which their individual averages have been subtracted (i.e., detrended). (c) A close-up from panel b. Transients (departure from the increasing trend/horizon), described in the manuscript, are shown by vertical black arrows. These transients are similar to observations related to the 1995 event at the Bio9 vent (1, 2), with some of them affecting multiple vents.

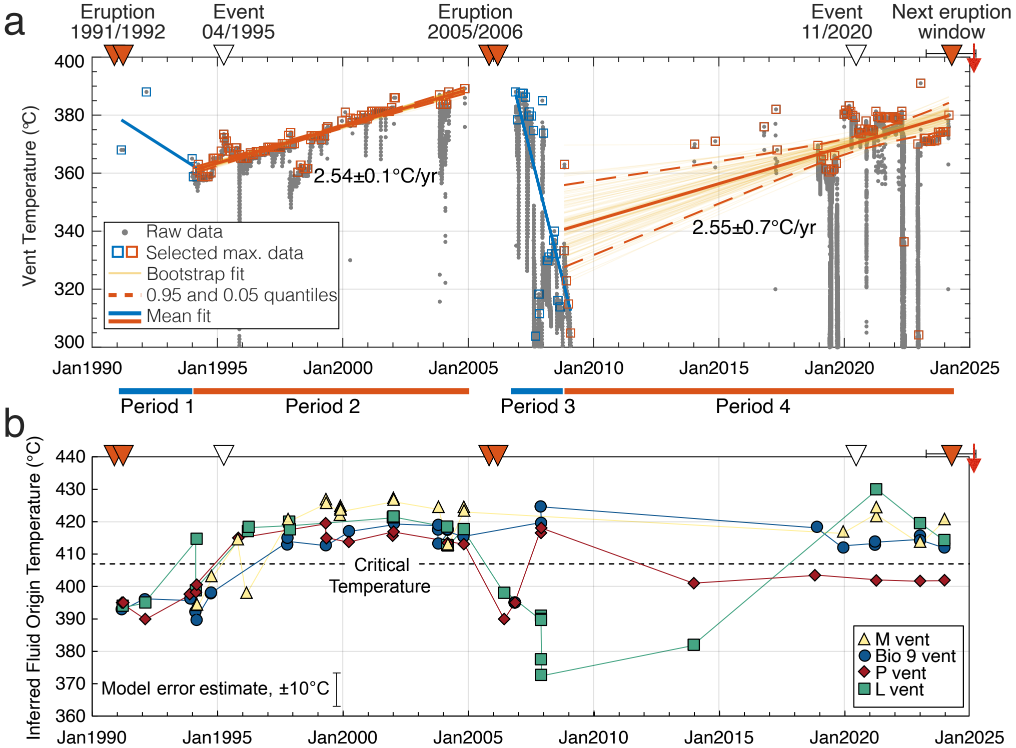

Fig. S4. (a) same as Fig. 2a. (b) Calculated origin temperatures for hydrothermal fluids from the EPR 9° 45'-54'N sampled between 1991 and 2024 are shown along with relationships to pre- and post-volcanic eruption periods. See Supporting Information Text below for further details on the geochemistry and geothermobarometric calculations for historic on-axis fluids.

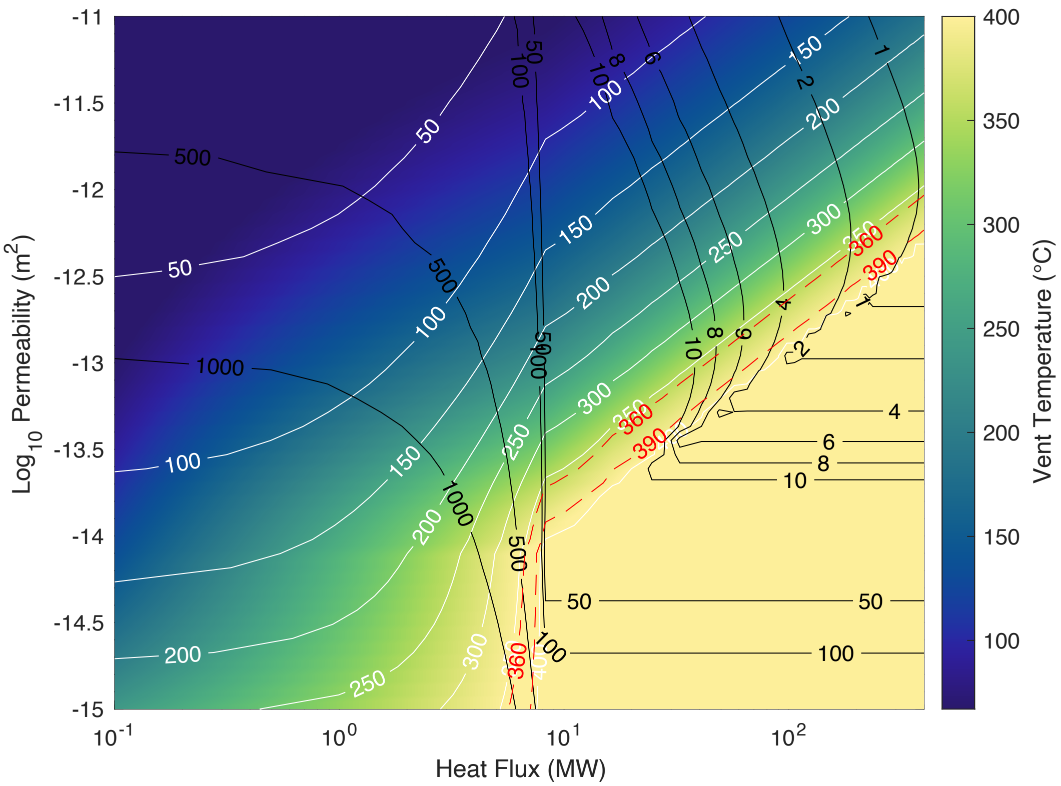

Fig. S5. Extended version of Fig. 3 in main manuscript to show hydrothermal vent temperatures (°C) and advection times (years, black solid lines) computed for low heat fluxes (MW) and crustal permeabilities (m^2) values within the zone of fluid flow. White solid lines are vent temperature contours. Red dashed lines indicate the EPR Bio9 vent temperature range during period 2, where exit fluid temperatures increase from 360°C to 390°C.

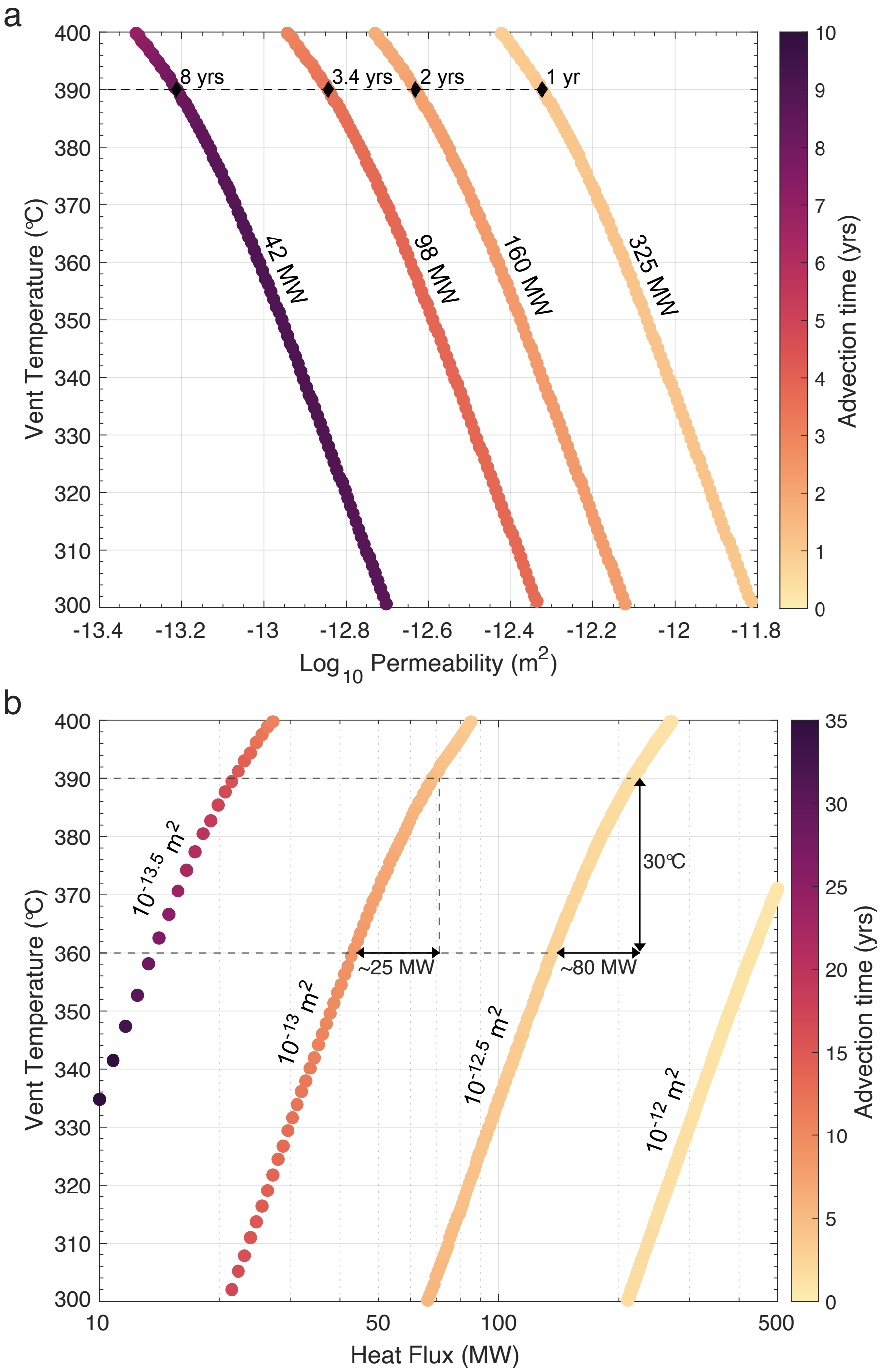

Fig. S6. (a) Advection time in years as a function of permeability *k* (log10(k) in m^2) and hydrothermal fluid temperature (°C), for given heat flux in MW (curve labels) calculated over an area of $\pi*R^2$, where r is the radius of the upflow zone, 50 m used here. As examples, hydrothermal fluid advection times are indicated as black diamonds for a 390°C fluid. Hydrothermal fluids would rise from the reaction zone to the seafloor in ~3.4 years for a hydrothermal vent field of 98 MW discharging 390°C vent fluids. (b) Advection time in years as a function of heat (MW) transported advectively (over an area of $\pi*R^2$, where r is the radius of the upflow zone, 50 m used here) and hydrothermal fluid temperature (°C), for given permeabilities (curve labels). For a hydrothermal system with a given constant permeability of 10^{-13} m^2, a temperature increase of 30°C corresponds to an increase of heat flux of about 25 MW. All curves were computed from Eq. (1) using a value of R = 50 m (half width of hydrothermal plume) and for fluid properties at 30 MPa.

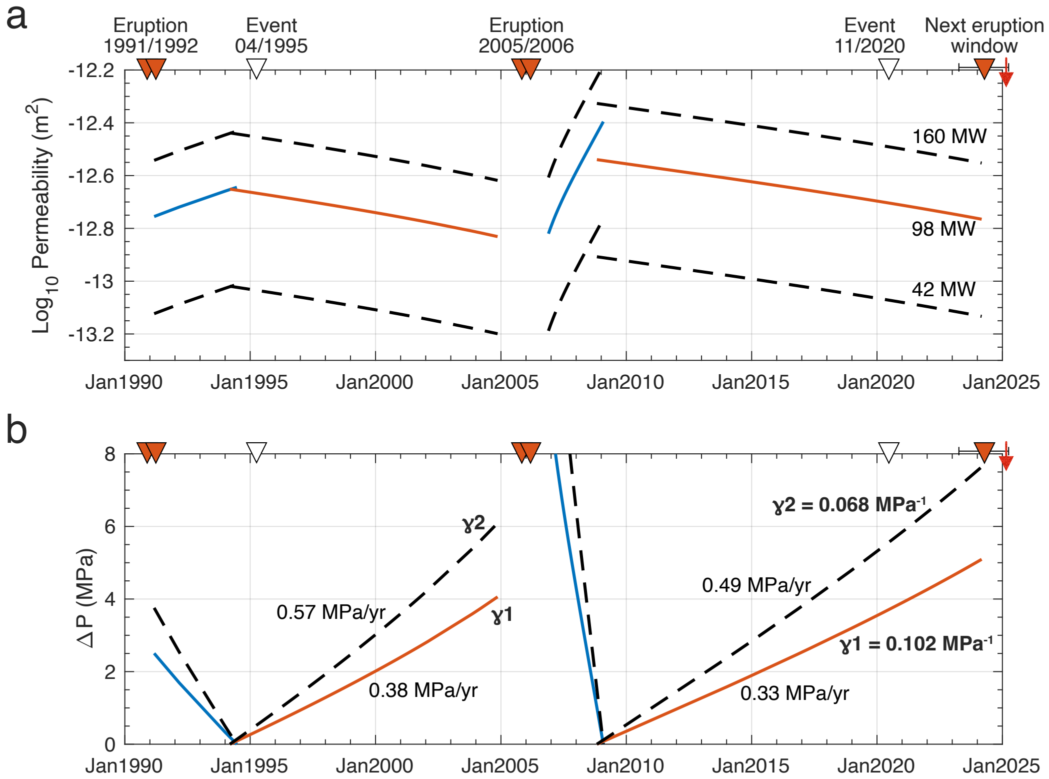

Fig. S7. (a) Temperature-derived permeability calculated over time for a given heat flux (curve labels). Note that different heat fluxes lead to different absolute permeability, however, the slopes (reflecting relative permeability changes over time) are the same. (b): Permeability-derived overpressure calculated over time for a given coefficient gamma (curve labels). Note that for a given gamma value, pressure changes over time for both periods 2 and 4 (corresponding to increasing temperature periods) are about the same. Both gamma values of 0.102 MPa/yr and 0.068 MPa/yr correspond to basalt rock type, from Kola and from Etna, respectively (4).

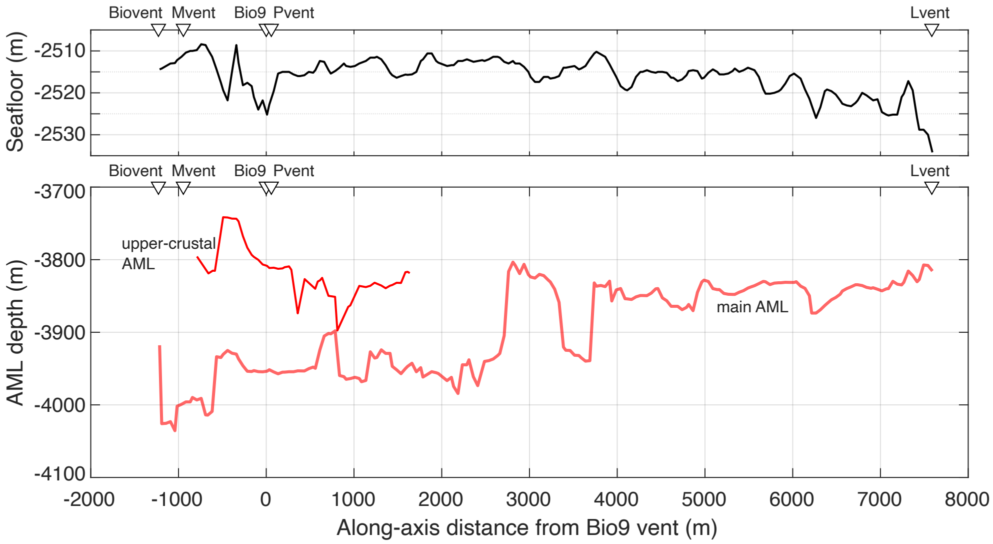

Fig. S8. (top panel) Along-axis (within the AST) bathymetric profile. (bottom panel) Corresponding along-axis axial magma lens (AML) depth profile in meters below sea-level – extracted from multichannel seismic (MCS) 3-D data displayed in Figs. 1c and 5a.

Supporting Information Text for Inferred Fluid Origin Temperatures Presented in Fig. S4b.

Fluid geochemistry methods

High-temperature hydrothermal fluids were sampled from M, Bio 9, P, and L vents between 1991-2024. In 1991-2007, fluids were collected by Karen Von Damm in titanium 'majors' samplers using the HOV *Alvin*. Fluids were stored in acid-washed high-density polyethylene (HDPE) bottles after acidification at sea with double-distilled 6 N HCl to pH <2. In the shore-based Von Damm laboratory, samples were filtered through 0.45 μm Nucleopore filters. Aqueous Mg abundance was determined via flame atomic absorption spectrophotometry (1991-2007; 5-9), while Fe and Mn abundances were determined via colorimetric analysis or flame atomic absorption spectrophotometry (1991-2004; 5) and via inductively-coupled plasma mass spectrometry (ICP-MS; 2004-2007; 6-9). In 2014, fluids were collected by Jeffrey Seewald in Seewald isobaric gastight samplers using the ROV *Jason*. In 2018-2019, fluids were collected by Daniel Fornari using the HOV *Alvin*. In 2021-2014, fluids were collected by Jill McDermott in Seewald isobaric gastight samplers using the ROV *Jason* (2021) and HOV *Alvin* (2023, 2024). Samples collected in 2014-2024 were analyzed via the following approach. One fluid aliquot was stored unacidified and unfiltered, in acid-washed HDPE bottles and analyzed via ion chromatography for Mg abundance. One fluid aliquot was acidified with Optima HCl to pH <2, in acid-washed HDPE bottles. In the shore-based McDermott laboratory, samples were filtered through 0.22 μm polyethersulfone filters. Filtered particles ('bottle solids') and solids rinsed from the sampler at sea ('dregs') articles were collected on Nylon filters and digested at 80°C in reverse aqua regia (1:3 Optima HCl:HNO_3). These three aliquots—aqueous, bottle solids, and dregs—were individually measured for Fe and Mn abundance via ICP-MS, and the full metals budget was mathematically reconstituted for each sample, for each metal.

Fluid endmember metal composition calculations

Studies indicate that high-temperature vent fluids undergo near-complete removal of dissolved Mg during circulation through the oceanic crust (e.g., 10). Therefore, for each discrete orifice at the M, Bio 9, P, and L vents, an 'endmember' composition for each date was determined by linearly regressing the concentrations of individual chemical species to zero Mg abundance. Endmember Fe and Mn abundances are presented in Table S1.

Geothermobarometric calculations for historic on-axis fluids

Origin temperatures shown in Fig. S4b were inferred for each vent, at each sampled interval, by dividing them into two groups: [1] fluids collected during non-eruptive periods (1993-2004, 2007-2024), and [2] fluids collected in post-eruptive periods (1991-1992 and 2006). Origin temperatures are not shown when trace fluid formation conditions approached the critical temperature of seawater (407 °C, 11), where small changes in pressure and temperature can drive large shifts in fluid composition and physical properties (12). Because this sensitivity in thermodynamic properties limits the accuracy of geothermobarometric estimates, origin temperatures within ±5 °C of the seawater critical temperature are not reported in this study.

For Bio9 and P vents in 1991 and 1992, post-eruption vapor-phase origin temperatures were taken from Von Damm (13). For L vent in 1991, 1992, and 2006 and for Bio9 and P in 2006, origin temperatures were estimated from endmember Cl concentrations reported in the legacy data set (7, 8) using the phase relationships of Driesner and Heinrich (14) and applying the same assumptions as Von Damm (13), including the assumption that fluids formed at pressures and temperatures below the seawater critical point (298.5 bar, 407 °C (11)). In 2006, measured venting temperatures were 383-388 °C at Bio9, 386-392 °C at P. These values are near the 391 °C two-phase boundary for seawater at the seafloor at 9° 50′ N EPR. This observation supports the interpretation that post-eruption fluids form just beneath the seafloor above a shallow heat source, similar to conditions in 1991-1992 (13). Estimated origin temperatures for post-eruption fluids have an uncertainty of ±8 °C, based on the resolution of the Driesner and Heinrich data (14).

Origin temperatures for non-eruptive fluids (1993-2004, 2007-2024) were determined using the Fe/Mn geothermometer, with an uncertainty of ±11 °C (15). This approach is based on the strong, temperature-dependent solubility of Fe and Mn during basalt alteration. The Fe/Mn abundance ratio is assumed to reflect the last temperature of fluid-mineral equilibration and is relatively insensitive to pressure and salinity variations.

Table S1.

Vent	Date	Endmember Fe mmol/kg	Endmember Mn µmol/kg	Reference
M Vent	29/02/1992	1,27	234	McDermott et al., 2022
M Vent	13/03/1992	1,63	288	McDermott et al., 2022
M Vent	10/03/1994	3,06	840	McDermott et al., 2022
M Vent	30/03/1994	3,08	843	McDermott et al., 2022
M Vent	29/10/1994	4,09	936	McDermott et al., 2022
M Vent	14/11/1995	5,30	961	McDermott et al., 2022
M Vent	21/03/1996	3,80	966	McDermott et al., 2022
M Vent	15/11/1997	5,64	900	McDermott et al., 2022
M Vent	20/05/1999	5,90	829	McDermott et al., 2022
M Vent	25/05/1999	5,69	819	McDermott et al., 2022
M Vent	09/12/1999	5,66	880	McDermott et al., 2022
M Vent	15/12/1999	5,93	870	McDermott et al., 2022
M Vent	18/12/1999	5,90	880	McDermott et al., 2022
M Vent	20/12/1999	5,84	878	McDermott et al., 2022
M Vent	24/01/2002	6,27	875	McDermott et al., 2022
M Vent	30/01/2002	6,20	875	McDermott et al., 2022
M Vent	11/11/2003	6,05	893	McDermott et al., 2022
M Vent	22/03/2004	3,98	895	McDermott et al., 2022
M Vent	28/03/2004	4,82	900	McDermott et al., 2022
M Vent	15/11/2004	6,03	890	McDermott et al., 2022
M Vent	21/11/2004	5,93	898	McDermott et al., 2022
M Vent	27/12/2019	5,53	954	This study
M Vent-South	20/04/2021	5,55	822	This study
M Vent-North	10/01/2024	5,71	911	This study
M Vent-North	20/01/2023	4,81	886	This study
M Vent-North	20/04/2021	6,31	988	This study
Bio9 Vent	01/04/1991	2,19	285	McDermott et al., 2022
Bio9 Vent	06/03/1992	1,67	172	McDermott et al., 2022
Bio9 Vent	28/12/1993	1,06	280	McDermott et al., 2022
Bio9 Vent	10/03/1994	1,28	367	McDermott et al., 2022
Bio9 Vent	29/03/1994	1,43	432	McDermott et al., 2022
Bio9 Vent	19/10/1994	2,31	590	McDermott et al., 2022
Bio9 Vent	24/10/1994	2,43	618	McDermott et al., 2022
Bio9 Vent	25/11/1995	6,03	1190	McDermott et al., 2022
Bio9 Vent	04/11/1997	4,95	929	McDermott et al., 2022
Bio9 Vent	10/11/1997	5,10	917	McDermott et al., 2022
Bio9 Vent	21/05/1999	3,63	685	McDermott et al., 2022
Bio9 Vent	20/04/2000	3,61	622	McDermott et al., 2022
Bio9 Vent	04/02/2002	2,68	441	McDermott et al., 2022
Bio9 Vent	10/11/2003	3,34	569	McDermott et al., 2022

Bio9 Vent	13/11/2003	2,82	525	McDermott et al., 2022
Bio9 Vent	13/11/2003	3,44	570	McDermott et al., 2022
Bio9 Vent	25/03/2004	3,42	585	McDermott et al., 2022
Bio9 Vent	27/03/2004	3,43	584	McDermott et al., 2022
Bio9 Vent	13/11/2004	3,32	593	McDermott et al., 2022
Bio9 Vent	24/11/2006	3,64	148	McDermott et al., 2022
Bio9 Vent	01/12/2006	3,46	130	McDermott et al., 2022
Bio9 Vent	11/12/2007	2,65	391	McDermott et al., 2022
Bio9 Vent	17/12/2007	2,41	395	McDermott et al., 2022
Bio9 Vent	12/01/2014	1,74	380	J. Seewald, pers. comm.
Bio9 Vent-Central	10/12/2018	1,40	230	This study
Bio9 Vent-Central	30/12/2019	2,34	392	This study
Bio9 Vent-Central	05/04/2021	1,90	364	This study
Bio9 Vent-Central	20/01/2023	2,03	374	This study
Bio9 Vent-Central	10/01/2024	2,04	373	This study
Bio9 Vent-South	05/04/2021	1,42	275	This study
Bio9 Vent-South	20/01/2023	2,17	407	This study
Bio9 Vent-South	10/01/2024	2,49	442	This study
P Vent	07/04/1991	4,42	175	McDermott et al., 2022
P Vent	23/04/1991	5,87	142	McDermott et al., 2022
P Vent	04/03/1992	0,0639	70,9	McDermott et al., 2022
P Vent	09/03/1992	0,687	70,1	McDermott et al., 2022
P Vent	18/12/1993	1,70	436	McDermott et al., 2022
P Vent	18/03/1994	2,89	732	McDermott et al., 2022
P Vent	27/03/1994	3,04	735	McDermott et al., 2022
P Vent	25/10/1994	5,39	1140	McDermott et al., 2022
P Vent	16/11/1995	7,10	1460	McDermott et al., 2022
P Vent	24/11/1995	8,28	1490	McDermott et al., 2022
P Vent	28/11/1995	7,49	1470	McDermott et al., 2022
P Vent	11/04/1996	6,82	1430	McDermott et al., 2022
P Vent	22/04/1996	6,81	1410	McDermott et al., 2022
P Vent	11/11/1997	6,02	1250	McDermott et al., 2022
P Vent	14/11/1997	6,03	1280	McDermott et al., 2022
P Vent	16/12/1997	5,37	1200	McDermott et al., 2022
P Vent	19/12/1997	6,00	1240	McDermott et al., 2022
P Vent	16/05/1999	6,53	1070	McDermott et al., 2022
P Vent	23/05/1999	6,21	1230	McDermott et al., 2022
P Vent	28/05/1999	6,13	1100	McDermott et al., 2022
P Vent	12/12/1999	6,57	1250	McDermott et al., 2022
P Vent	17/04/2000	6,82	1260	McDermott et al., 2022
P Vent	16/01/2002	7,25	1290	McDermott et al., 2022
P Vent	30/01/2002	7,40	1280	McDermott et al., 2022
P Vent	09/11/2003	6,58	1260	McDermott et al., 2022
P Vent	13/11/2003	6,59	1250	McDermott et al., 2022

P Vent	24/03/2004	5,67	1210	McDermott et al., 2022
P Vent	27/03/2004	6,53	1210	McDermott et al., 2022
P Vent	17/11/2004	6,38	1190	McDermott et al., 2022
P Vent	27/06/2006	0,802	79,9	McDermott et al., 2022
P Vent	28/11/2006	1,08	92,2	McDermott et al., 2022
P Vent	01/12/2006	1,31	96,7	McDermott et al., 2022
P Vent	12/12/2007	2,90	506	McDermott et al., 2022
P Vent	17/12/2007	3,10	524	McDermott et al., 2022
P Vent	03/01/2014	1,56	377	J. Seewald, pers. comm.
P Vent	10/12/2018	1,80	409	This study
P Vent	30/12/2019	2,73	576	This study
P Vent	20/04/2021	1,94	456	This study
P Vent	20/01/2023	1,86	440	This study
P Vent	10/01/2024	2,08	489	This study
L Vent	17/04/1991	1,72	109	K. L. Von Damm, unpubl.
L Vent	10/03/1992	2,09	163	K. L. Von Damm, unpubl.
L Vent	16/03/1994	5,07	1270	K. L. Von Damm, unpubl.
L Vent	26/03/1994	7,29	1320	K. L. Von Damm, unpubl.
L Vent	10/04/1996	8,47	1460	K. L. Von Damm, unpubl.
L Vent	24/04/1996	8,58	1440	K. L. Von Damm, unpubl.
L Vent	03/12/1997	8,32	1350	K. L. Von Damm, unpubl.
L Vent	14/12/1997	7,51	1280	K. L. Von Damm, unpubl.
L Vent	17/01/2002	7,12	1130	K. L. Von Damm, unpubl.
L Vent	31/01/2002	7,13	1120	K. L. Von Damm, unpubl.
L Vent	20/03/2004	7,04	1179	K. L. Von Damm, unpubl.
L Vent	29/03/2004	6,72	1270	K. L. Von Damm, unpubl.
L Vent	14/11/2004	6,61	1120	Bryce et al., 2015a
L Vent	29/06/2006	0,405	61,4	Bryce et al., 2015b
L Vent	26/11/2006	4,25	657	Bryce et al., 2015c
L Vent	13/12/2007	1,35	525	Bryce et al., 2015d
L Vent	18/12/2007	1,33	571	This study
L Vent	16/01/2014	1,17	413	J. Seewald, pers. comm.
L Vent	20/01/2023	4,37	717	This study
L Vent	10/01/2024	4,07	743	This study
L-Hot8 Vent	31/01/2002	5,76	1546	K. L. Von Damm, unpubl.
L-Hot8 Vent	29/03/2004	6,08	1639	K. L. Von Damm, unpubl.
L-Hot8 Vent	13/12/2007	1,85	540	Bryce et al., 2015d
L-Hot8 Vent	18/12/2007	1,83	546	Bryce et al., 2015d
L-Mkr22 Vent	31/01/2002	7,54	1440	K. L. Von Damm, unpubl.
L-Mkr22 Vent	23/03/2004	7,99	1510	K. L. Von Damm, unpubl.
L-Mkr22 Vent	26/11/2006	3,28	355	Bryce et al., 2015c
L-Mkr22 Vent	04/12/2006	3,53	366	Bryce et al., 2015c
L-Mkr22 Vent	18/12/2007	2,34	708	Bryce et al., 2015d
L-Hot8-diff Vent	20/04/2021	4,91	650	This study

SI References

1. R.A. Sohn, D.J. Fornari, K.L. Von Damm, J.A. Hildebrand, & S.C. Webb, Seismic and hydrothermal evidence for a cracking event on the East Pacific Rise crest at 9 50′ N, *Nature* 396, 159-161 (1998).
2. D.J. Fornari, T. Shank, K.L. Von Damm, T.K.P. Gregg, M. Lilley, G. Levai, A. Bray, R.M. Haymon, M.R. Perfit, and R. Lutz, Time-series temperature measurements at high-temperature hydrothermal vents, East Pacific Rise 9 49′–51′ N: Evidence for monitoring a crustal cracking event. *Earth and Planetary Science Letters*, *160*(3-4), pp.419-431 (1998).
3. M. Wei-Haas, "Volcanic Eruption in Deep Ocean Ridge Is Witnessed by Scientists for First Time" (2025); https://www.nytimes.com/2025/05/02/science/deep-ocean-volcanic-eruption-pacific.html
4. A. Ougier-Simonin, Y. Guéguen, J. Fortin, A. Schubnel, & F. Bouyer, Permeability and elastic properties of cracked glass under pressure. *Journal of Geophysical Research: Solid Earth*, *116*(B7) (2011).
5. K. L. Von Damm, J. M. Edmond, B. Grant, C. I. Measures, B. Walden, R. F. Weiss, *Chemistry of submarine hydrothermal solutions at 21° N, East Pacific Rise. Geochim. Cosmochim. Acta* 49, 2197–2220 (1985)
6. J. Bryce, F. Prado, K. Von Damm, Vent Fluid Chemistry Data from samples acquired with HOV Alvin during Atlantis expedition AT11-20 2004 at the East Pacific Rise 9N site. IEDA. (2015a) doi:10.1594/IEDA/317362
7. J. Bryce, F. Prado, K. Von Damm, Vent Fluid Chemistry Data, including Metals Fractions and Dissolved Majors, from fluid samples acquired with HOV Alvin during Atlantis expedition AT15-06 (2006) at the East Pacific Rise 9N site. IEDA. (2015b) doi:10.1594/IEDA/317365
8. J. Bryce, F. Prado, K. Von Damm, Vent Fluid Chemistry Data, including Metals Fractions and Dissolved Majors, from fluid samples acquired with HOV Alvin during Atlantis expedition AT15-13 (2006) at the East Pacific Rise 9N site. IEDA. (2015c) doi:10.1594/IEDA/317364
9. J. Bryce, F. Prado, K. Von Damm, Vent Fluid Chemistry Data, including Metals Fractions and Dissolved Majors, from fluid samples acquired with HOV Alvin during Atlantis expedition AT15-27 (2007) at the East Pacific Rise 9N site. IEDA. (2015d) doi:10.1594/IEDA/317603
10. J. L. Bischoff, F. W. Dickson, Seawater-basalt interaction at 200°C and 500 bars: Implications for origin of seafloor heavy-metal deposits and regulation of seawater chemistry. *Earth Planet. Sci. Lett.* 25, 385–397 (1975).
11. J. L. Bischoff, R. J. Rosenbauer, Liquid-vapor relations in the critical region of the system NaCl-H_2O from 380 to 415°C: A refined determination of the critical point and two-phase boundary of seawater. *Geochim. Cosmochim. Acta,* 52, 2121-2126 (1988).
12. E. L. Shock Chemical environments of submarine hydrothermal systems. *Origins Life Evol. Biosphere* 22, 67–107 (1992).
13. K. L. Von Damm, Evolution of the hydrothermal system at East Pacific Rise 9°50'N: Geochemical evidence for changes in the upper oceanic crust. *Geophys. Monogr. Ser.* 148, 285–304 (2004).
14. T. Driesner, C. A. Heinrich, The system H_2O–NaCl. Part I: Correlation formulae for phase relations in temperature–pressure–composition space from 0 to 1000°C, 0 to 5000bar, and 0 to 1 XNaCl, *Geochim. Cosmochim. Acta*, 71(20), 4880–4901 (2007).
15. N. J. Pester, M. Rough, K. Ding, W. E. Seyfried, A new Fe/Mn geothermometer for hydrothermal systems: Implications for high-salinity fluids at 13°N on the East Pacific Rise. *Geochim. Cosmochim. Acta.* 75, 7881–7892 (2011).
16. J. M. McDermott, R. Parnell-Turner, T. Barreyre, S. Herrera, C. C. M. Downing, N. Pittoors, K. P. Pehr, S. A. Vohsen, W. S. D. Dowd, J.-N. Wu, M. Marjanović, D. J. Fornari, Discovery of active off-axis hydrothermal vents at 9° 54′ N East Pacific Rise. Proc. Natl. Acad. Sci. U.S.A. 119, e2205602119 (2022)
